# The Role of Serotonin in Concanavalin A-Induced Liver Injury in Mice

**DOI:** 10.1155/2020/7504521

**Published:** 2020-01-04

**Authors:** Qing Pang, Hao Jin, Xiquan Ke, Zhongran Man, Yong Wang, Yi Tan, Zheng Lu, Huichun Liu

**Affiliations:** ^1^Department of Hepatobiliary Surgery, First Affiliated Hospital of Bengbu Medical College, Bengbu, 233000 Anhui Province, China; ^2^Department of Hepatobiliary Surgery, First Affiliated Hospital of Xi'an Jiaotong University, Xi'an, 710061 Shaanxi Province, China; ^3^Department of Gastroenterology, First Affiliated Hospital of Bengbu Medical College, Bengbu, 233000 Anhui Province, China

## Abstract

Serotonin is involved in the pathological processes of several liver diseases via the regulation of inflammatory response and oxidative stress. We aimed to investigate the role of serotonin in Concanavalin A- (Con A-) induced acute liver injury (ALI). ALI was induced in C57B/6 wild-type (WT) mice and tryptophan hydroxylase 1 (TPH1) knockout mice through tail vein injection of Con A (15 mg/kg body weight). Another group of TPH1 knockout ALI mice was supplied with 5-hydroxytryptophan (5-HTP) in advance to recover serotonin. The blood and liver tissues of mice were collected in all groups. Markedly increased serum levels of serotonin were identified after the injection of Con A. Increased serum levels of alanine aminotransferase (ALT) and aspartate aminotransferase (AST) and stronger hepatic tissue pathology were detected, suggesting that serotonin could mediate Con A-induced liver damage. Serotonin significantly facilitated the release of serum and intrahepatic inflammatory cytokines, including interleukin-2 (IL-2), interleukin-6 (IL-6), interleukin-17A (IL-17A), interferon-gamma (IFN-*γ*), and tumor necrosis-alpha (TNF-*α*), after the administration of Con A. In addition, serotonin significantly increased the intrahepatic levels of oxidative stress markers malonaldehyde (MDA), myeloperoxidase (MPO), and nitric oxide (NO) and decreased antioxidant stress indicator glutathione (GSH) in Con A-treated mice. Additionally, serotonin promoted hepatocyte apoptosis and autophagy based on B-cell lymphoma-2 (Bcl-2), Bcl-2-asociated X protein (Bax), and Beclin-1 levels and TUNEL staining. More importantly, serotonin activated nuclear factor kappa B (NF-*κ*B) and upregulated the hepatic expressions of high mobility group protein B1 (HMGB1), toll-like receptor-4 (TLR4), and downstream molecules in Con A-mediated liver injury. Serotonin 2A receptor was upregulated in liver tissue after Con A injection, and serotonin 2A receptor antagonist Ketanserin protected against Con A-induced hepatitis. These results indicated that serotonin has the potential to aggravate Con A-induced ALI via the promotion of inflammatory response, oxidative stress injury, and hepatocyte apoptosis and the activation of hepatic HMGB1-TLR signaling pathway and serotonin 2A receptor.

## 1. Introduction

Acute liver injury (ALI) refers to a destructive hepatic inflammatory disease, which can be caused by virus infection, hepatotoxic drugs, alcohol abuse, and so forth [1, 2]. Severe and persistent ALI can eventually lead to liver failure, liver cirrhosis, and tumorigenesis [3]. To date, the pathological mechanism of ALI is not completely clear.

Concanavalin A (Con A) is a lectin-like polysaccharide derived from plants. In 1992, Tiegs et al. successfully established the mouse model of ALI by tail vein injection of Con A [4]. This model is accompanied by massive liver tissue necrosis, extensive inflammatory response, and prominent hepatocyte apoptosis [5]. In addition, remarkable oxidative stress injury, characterized by significant upregulation in contents of oxidative markers and significant downregulation in activities of antioxidative markers, is closely related to inflammation and Con A-mediated hepatocyte damage [5–8]. As easy operation, low cost, well repeatability, and close to the pathological process of human viral hepatitis, Con A-induced ALI is regarded as the best experimental model for ALI research [9, 10].

As one of the earliest identified pattern recognition receptors (PRRs), toll-like receptors (TLRs) have been widely studied and are involved in the regulation of inflammatory and apoptotic responses [11]. Previous studies have shown that Con A can stimulate the expression of TLRs and downstream pathways in liver tissue, leading to the initiation of inflammatory response [12, 13]. After binding ligand, TLRs stimulate nuclear factor kappa B (NF-*κ*B) activation and trigger the release of various inflammatory mediators, such as tumor necrosis-alpha (TNF-*α*), interleukin-6 (IL-6), and interleukin-17 (IL-17) [11, 14].

Serotonin, also known as 5-hydroxytryptophan (5-HT), is a kind of indoleamine compound widely distributed in all kinds of mammals. Serotonin is mainly divided into central and peripheral types. The latter type, which is mainly stored in dense granules of platelets after being released from intestinal enterochromaffin cells, accounts for approximately 95% of serotonin. Previous studies have shown that peripheral serotonin participates in platelet aggregation, gastrointestinal peristalsis, oxidative stress injury, inflammation and immune response, and so forth [15–21]. In addition, peripheral serotonin may be involved in the progress of several liver diseases such as liver regeneration, viral hepatitis, nonalcoholic steatohepatitis, hepatic fibrosis, and hepatocellular carcinoma via binding to 5-HT_2A_ and 2B receptors in liver tissue [22–25]. However, the role of peripheral serotonin in Con A-induced ALI remains unclear. In this study, we aimed to investigate the role of serotonin in Con A-mediated liver injury.

## 2. Materials and Methods

### 2.1. Experimental Animals

Male wild-type (WT) C57BL/6 mice (purchased from Animal Feeding Center of Xi'an Jiaotong University Health Science Center, Xi'an, China) and male mice with TPH1 (critical rate-limiting enzyme for the synthesis of peripheral serotonin) knockout (TPH1−/−, which originated from C57BL/6 mice, kindly presented by Max Planck Institute for Molecular Genetics, Berlin, Germany, and the genotype was tested by PCR) were used (6-8 weeks old, 20-25 g). As previously described, the lack of peripheral serotonin is the only difference between TPH1−/− and WT mice [26]. All mice were housed in pathogen-free polycarbonate cages at a constant temperature of 25°C and a humidity of 50% with 12 h light-dark cycles. All mice were fed with standard diet and water ad libitum. Animal care was in compliance with the criteria of Guide for the Care and Use of Laboratory Animals established by the US National Institutes of Health. The study was approved by the Animal Research Committee of Xi'an Jiaotong University Health Science Center.

### 2.2. Experimental Groups and Animal Model of ALI

To construct an ideal ALI animal model, different dosages of Con A (8, 15, and 25 mg/kg body weight, *n* = 6 per group) were administered in advance in C57BL/6 WT mice to compare survival and hepatic pathological damage. With the suitable ALI model (15 mg/kg body weight of Con A), the mice were randomly allocated into the following groups (*n* = 12 per group): (1) WT (or TPH1-/-)+normal saline (NS, 0.9% sodium chloride injection, which is the solvent of Con A) groups: WT or TPH1-/- mice were treated with NS (0.2 ml/20 g body weight) through single tail vein injection; (2) WT (or TPH1-/-)+Con A group: WT or TPH1-/- mice were treated with Con A (Sigma-Aldrich, USA, 15 mg/kg body weight, suspended in NS in a final volume of 0.2 ml/20 g body weight) through single tail vein injection; (3) TPH1-/-+5-hydroxytryptophan (5-HTP)+Con A group: TPH1 knockout mice were treated with Con A (15 mg/kg body weight) and 5-HTP (precursor of serotonin, which can recover the serotonin content at a dose of 75 mg/kg/day for 3 days by subcutaneous injection before Con A administration [26]). At 24 h after the administration of Con A or NS, all mice were anesthetized with isoflurane gas and were sacrificed by cervical decapitation. Then, the abdominal skin was disinfected, abdominal cavity was opened, and about 100 mg of the left lobe of the liver was cut off for the extraction of protein and RNA. The rest of the left lobe was used for histological examination. In another series of experiments, according to the groups mentioned above, mice (*n* = 10 per group) were monitored for assessing mortality after administration of NS or a lethal dose of Con A (25 mg/kg body weight).

### 2.3. Liver Function Analysis

At 6 h and 24 h after the administration of Con A or NS, blood samples (*n* = 6) were collected and centrifuged at 4°C in tubes. 0.1 ml of serum was obtained and diluted to 0.5 ml with NS. Then, liver function was evaluated by alanine aminotransferase (ALT) and aspartate aminotransferase (AST) levels, which were determined by enzymatic colorimetric assay using an Olympus AU5400 Automatic Biochemical Analyzer (Olympus, Tokyo, Japan).

### 2.4. Histological Examination

Excised liver tissues (*n* = 6) were fixed in 10% formalin solution and were embedded in paraffin. Serial 5 *μ*m thickness sections were stained with Hematoxylin and Eosin (H&E) to evaluate the morphology. The results were examined by two independent researchers.

### 2.5. Measurement of Serum Inflammation Cytokines and Serotonin

At 6 h and 24 h after the administration of Con A or NS, serum levels of interleukin-2 (IL-2), interleukin-4 (IL-4), IL-6, interleukin-10 (IL-10), IL-17A, interferon-gamma (IFN-*γ*), and TNF-*α* were measured (*n* = 6) by using ELISA kits (Dakewe Biotech Company, Shenzhen, China). At 0 h, 6 h, 24 h, and 48 h after Con A or NS injection, serum level of serotonin was measured (*n* = 6) by using an ELISA kit (Dakewe Biotech Company, Shenzhen, China) according to the manufacturer's instructions.

### 2.6. Oxidative Stress Assay

The liver tissue was obtained and homogenized at 24 h after the administration of Con A or NS. Liver glutathione (GSH), malonaldehyde (MDA), myeloperoxidase (MPO), nitric oxide (NO), and total superoxide dismutase (SOD) were measured using the activity assay kits (Jiancheng Bioengineering Institute, Nanjing, China) according to the manufacturer's instructions.

### 2.7. Transferase-Mediated dUTP Nick End-Labeling (TUNEL) Assay

Hepatocyte apoptosis was detected by TUNEL method using the In Situ Cell Death Detection Kit (Jiancheng Bioengineering Institute, Nanjing, China) according to the manufacturer's instructions. Paraffin-embedded sections were firstly dewaxed in xylene and were then rehydrated through a graded series of ethanol solutions ending with phosphate-buffered saline. Sections were permeabilized with proteinase K (20 mg/ml in 10 mmol/l Tris-HCl, pH 7.4–8.0) at 37°C for 15 min. After washing, sections were treated with TdT and were then viewed and photographed using a common light microscope.

### 2.8. RNA Isolation and Quantitative Reverse Transcription-Polymerase Chain Reaction (qRT-PCR) Analysis

Liver tissue samples from each group were snap-frozen in liquid nitrogen and stored at -80°C until usage. Total RNA was isolated using the RNAfast200 Kit (Fastagen Biotech, Shanghai, China), and reverse transcription was performed using the PrimeScript RT reagent Kit (TaKaRa Biotechnology, Dalian, China). The mRNA expression was assayed in triplicate and normalized to GAPDH. The relative levels were calculated using the Comparative-Ct Method (DDCt method). The primers used in the study were listed in Supplementary [Supplementary-material supplementary-material-1]. The reaction condition of qRT-PCR is as follows: after predenaturation at 95°C for 2 min, the following was carried out for 40 cycles: denaturation at 94°C for 60 sec, annealing at 55°C for 40 sec, extension at 72°C for 60 sec, and fully extended at 72°C for 5 min in the last cycle.

### 2.9. Western Blot Assay

The monoclonal antibodies (Supplementary [Supplementary-material supplementary-material-1]) used in this research were purchased from Cell Signaling Technology (Beverly, MA, USA). Protein concentration was determined by the method of BCA. Western blot analysis was performed as previously described [26].

### 2.10. Statistical Analysis

SPSS 20.0 (SPSS, IBM Inc.) was used to analyze data. The mortality rates were expressed as percentage. Continuous data were expressed as mean ± standard deviation (SD). The differences between groups were compared by using Student's *t*-test, one-way analysis of variance (ANOVA), and LSD post hoc test, or Wilcoxon test, as applicable. Survival was estimated by the Kaplan-Meier (KM) curve with log-rank test. A *P* value less than 0.05 was considered statistically significant.

## 3. Results

The optimal dosage of Con A used in ALI model remains controversial [10]. To obtain the suitable ALI model, the preliminary experiments were administered to compare 24 h survival and hepatic pathological damage between different dosages of Con A. After modeling with a lower dose (8 mg/kg body weight) of Con A, none of the model mice was sacrificed (24 h survival rate: 100%), and mild hepatocyte necrosis was observed in the hepatic lobule. After injection with a medium Con A dose (15 mg/kg body weight), all mice survived (24 h survival rate: 100%), and there were obvious necrotic areas in the liver tissue accompanied by irregular hepatocyte structure and numerous inflammatory cell infiltrations. In contrast, there were 3 mice which were sacrificed (24 h survival rate: 50%) after injection with a higher dose of Con A (25 mg/kg body weight), and extensive necrosis areas and disorganized hepatocyte structure were observed in the remaining surviving mice (Supplementary [Supplementary-material supplementary-material-1]). Therefore, the dose of 15 mg/kg body weight Con A was selected for the subsequent experiments.

### 3.1. Serotonin Aggravated Liver Injury in Con A-Mediated ALI Mice

**Figure 1:**
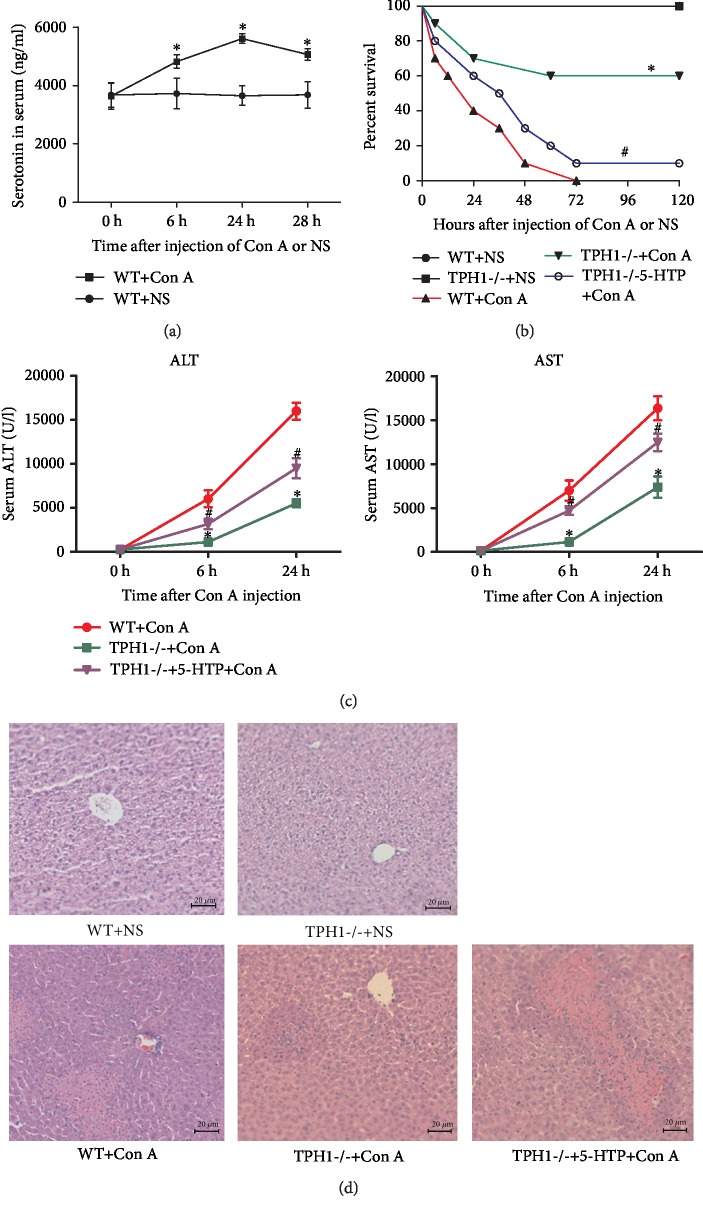
Serotonin aggravated liver injury in Con A-mediated ALI mice. (a) The changes of serum serotonin level in mice after the administration of Con A. ^∗^*P* < 0.05 compared with 0 h by paired *t*-test. (b) Comparison of 120 h survival after injection of a sublethal dose of Con A (25 mg/kg). ^∗^*P* < 0.05 compared with the WT+Con A group; ^#^*P* < 0.05 compared with the TPH1-/-+Con A group by log-rank test. (c) Comparison of serum ALT and AST in ALI mice. ^∗^*P* < 0.05 compared with the WT+Con A group at the same time point; ^#^*P* < 0.05 compared with the TPH1-/-+Con A group at the same time point by one-way ANOVA and LSD test. (d) H&E staining (×200) was performed on liver tissue slices at 24 h after the administration of Con A.

To preliminarily determine the role of serotonin on Con A-induced ALI, changes of serum serotonin levels were detected after the injection of Con A in WT mice. As shown in Figure 1(a), compared with pretreatment (3635.7 ± 365.8 ng/ml), the serum levels of serotonin significantly increased at 6 h (4328.8 ± 186.5 ng/ml, *P* = 0.006), 24 h (5610.5 ± 132.6 ng/ml, *P* < 0.001), and 48 h (5068.8 ± 165.6 ng/ml, *P* = 0.003) after Con A administration. In contrast, there were no significant changes in the levels of serotonin after administration of NS (Figure 1(a)). It was suggested that serotonin may play a role in Con A-induced ALI. We also detected the serum baseline levels of serotonin (without the administration of Con A or NS) in WT and TPH1-/- mice with or without the supplement of 5-HTP. Compared with WT mice, the basal level of serotonin in TPH1-/- mice was remarkably decreased (99.5 ± 8.9 ng/ml, *P* < 0.001). However, the supplement of 5-HTP in TPH1-/- mice significantly restored serotonin (2765.1 ± 308.5 ng/ml, *P* < 0.001). In contrast, the addition of 5-HTP in WT mice slightly elevated the serotonin level, but there was no statistical significance (Supplementary [Supplementary-material supplementary-material-1]).

Then, we explored the effect of serotonin on the survival of mice with a sublethal dose of Con A. The 120 h survival rate in the TPH1-/- model group was significantly higher than that in the WT model group (Figure 1(b), 60% *vs.* 0%, *χ*^2^ = 8.355, *P* = 0.004). However, the supplement of 5-HTP significantly decreased survival in TPH1-/- model mice. These results indicated that serotonin may have an adverse effect on Con A-induced ALI.

Serum levels of ALT and AST were detected to assess the degree of liver injury. Compared with the WT model group, levels of ALT and AST were significantly improved in the TPH1-/- model group at 6 h (ALT: 1103.9 ± 611.10*vs*. 6015.4 ± 2139.0 U/l, *P* = 0.001; AST: 1134.5 ± 876.8*vs*. 6992.9 ± 2559.2 U/l, *P* < 0.001) and at 24 h (ALT: 5511.5 ± 1036.8*vs*. 15986.9 ± 2132.3 U/l, *P* = 0.005; AST: 7381.7 ± 2691.0*vs*. 16352.1 ± 3025.1 U/l, *P* = 0.001) after the injection of Con A (Figure 1(c)). Moreover, ALT and AST levels in TPH1-/- model mice with the pretreatment of 5-HTP were significantly higher than those without pretreatment. The histopathological examination was further performed, and hepatocyte degeneration and obvious necrotic areas were observed in mice of Con A-induced hepatitis. The TPH1-/- model group showed minor histological damage compared with the WT model group (Figure 1(d)). In contrast, TPH1-/- mice treated with 5-HTP presented a more serious pathological damage, indicating that serotonin significantly aggravated hepatic pathological injury and hepatocyte necrosis in Con A-induced ALI.

### 3.2. Effect of Serotonin on the Release of Serum Inflammatory Cytokines in Con A-Treated Mice

**Figure 2:**
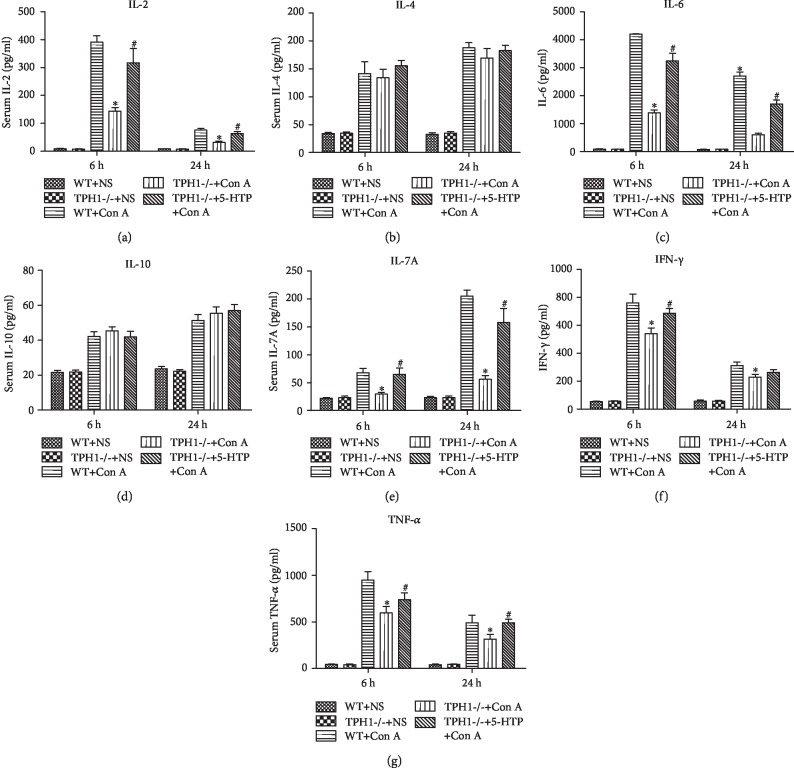
Serotonin facilitated the release of serum inflammatory cytokines in Con A-mediated ALI mice. The levels of (a) IL-2, (b) IL-4, (c) IL-6, (d) IL-10, (e) IL-17A, (f) IFN-*γ*, and (g) TNF-*α* in serum were assessed by ELISA.^∗^*P* < 0.05 compared with the WT+Con A group at the same time point; ^#^*P* < 0.05 compared with the TPH1-/-+Con A group at the same time point by one-way ANOVA and LSD test.

Inflammatory response plays an essential role in the pathogenesis of Con A-induced ALI [6, 27]. As shown in Figure 2, Con A stimulated the release of several inflammatory cytokines, including IL-2, IL-4, IL-6, IL-10, IL-17A, IFN- *γ*, and TNF- *α*, to different extents. Compared with the WT model group, the serum levels of proinflammatory cytokines IL-2 (6 h: 144.3 ± 28.1*vs*. 390.8 ± 52.0 pg/ml, *P* < 0.001; 24 h: 32.1 ± 9.4*vs*. 76.1 ± 14.3 pg/ml, *P* < 0.001), IL-6 (6 h: 4200.6 ± 28.1*vs*. 1376.7 ± 240.5 pg/ml, *P* < 0.001; 24 h: 2700.2 ± 329.1*vs*. 600.9 ± 104.2 pg/ml, *P* < 0.001), IL-17A (6 h: 67.6 ± 18.3*vs*. 29.5 ± 7.1 pg/ml, *P* = 0.002; 24 h: 204.8 ± 25.1*vs*. 55.9 ± 15.5 pg/ml, *P* < 0.001), IFN- *γ* (6 h: 760.7 ± 138.3*vs*. 540.6 ± 91.1 pg/ml, *P* = 0.018; 24 h: 310.9 ± 58.7*vs*. 228.5 ± 46.0 pg/ml, *P* = 0.039), and TNF- *α* (6 h: 950.3 ± 202.5*vs*. 595.4 ± 159.1 pg/ml, *P* = 0.015; 24 h: 491.4 ± 180.7*vs*. 315.0 ± 111.9 pg/ml, *P* = 0.101) significantly decreased in the TPH1-/- model group. Moreover, the recovery of serotonin in TPH1-/- model mice reversed the above changes. However, there were no significant differences in the levels of IL-4 and IL-10 between the three model groups.

### 3.3. Effect of Serotonin on Intrahepatic mRNA Expression of Inflammatory Cytokines in Con A-Treated Mice

**Figure 3:**
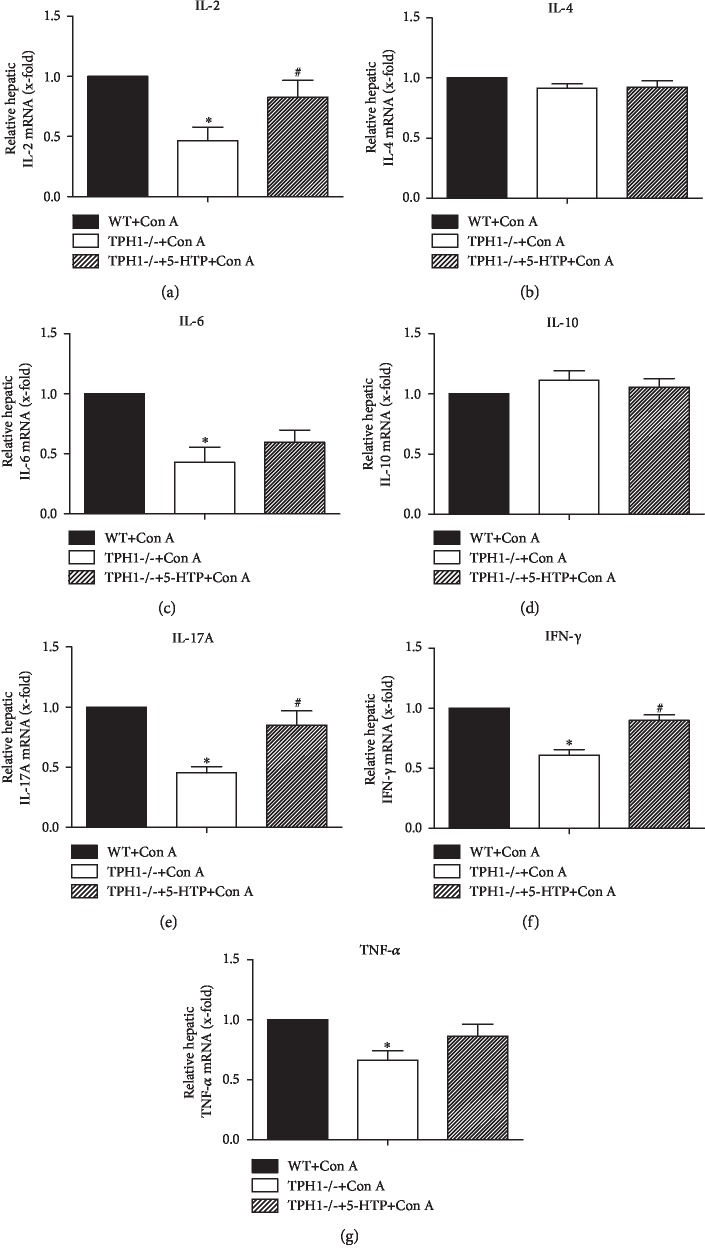
Serotonin induced the release of intrahepatic inflammatory cytokines in Con A-mediated ALI mice. The mRNA levels of (a) IL-2, (b) IL-4, (c) IL-6, (d) IL-10, (e) IL-17A, (f) IFN-*γ*, and (g) TNF-*α* in liver tissue 24 h after the administration of Con A were assessed by qRT-PCR. ^∗^*P* < 0.05 compared with the WT+Con A group; ^#^*P* < 0.05 compared with the TPH1-/-+Con A group by one-way ANOVA and LSD test.

After the administration of Con A, numerous inflammatory cells are recruited to the liver and produce various hepatotoxic cytokines, which ultimately lead to inflammatory liver injury. We found that the mRNA expressions of proinflammatory IL-2 (*P* = 0.002), IL-6 (*P* = 0.002), IL-17A (*P* < 0.001), IFN- *γ* (*P* < 0.001), and TNF- *α* (*P* = 0.003) in liver tissue were significantly lower in the TPH1-/- model group than those in the WT model group (Figure 3, *P* < 0 05). In contrast, the supplement of 5-HTP upregulated the intrahepatic expressions of IL-2, IL-17A, and IFN-*γ* after Con A injection. However, there were no obvious differences in the intrahepatic mRNA expressions of IL-4 and IL-10 between the three model groups. Therefore, these results provided strong evidence that serotonin facilitated the release of proinflammatory cytokines in Con A-mediated liver injury.

### 3.4. Effect of Serotonin on Hepatic Oxidative Stress Injury in Con A-Treated Mice

**Figure 4:**
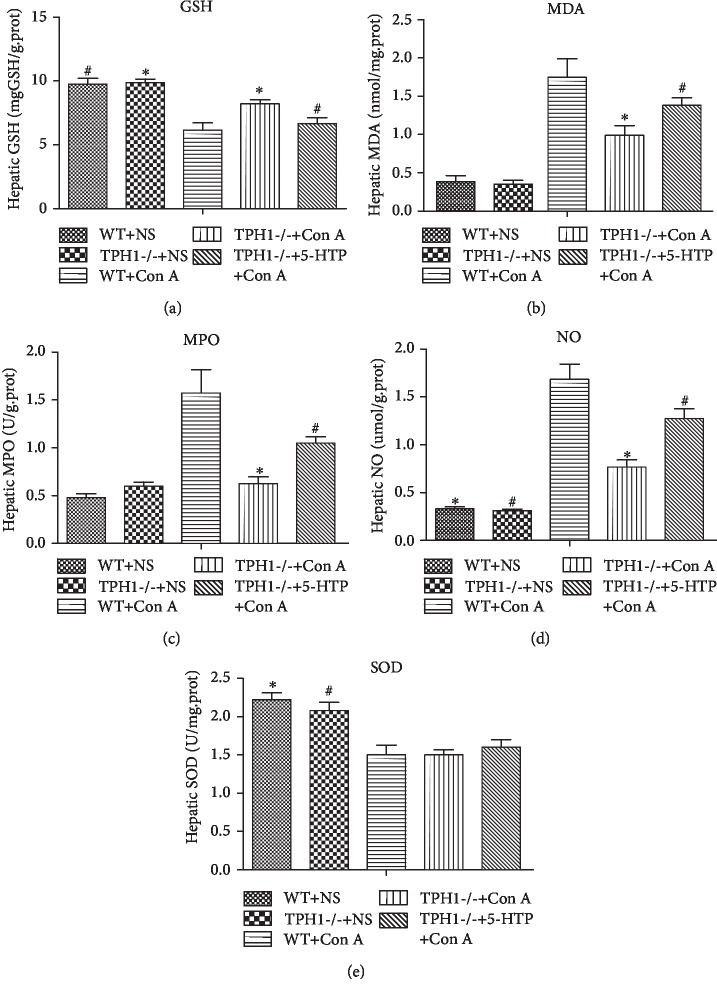
Serotonin exacerbated oxidative stress-mediated hepatocellular injury in Con A-mediated ALI mice. The levels of (a) GSH, (b) MDA, (c) MPO, (d) NO, and (e) SOD in liver tissue were assessed at 24 h after the administration of Con A. ^∗^*P* < 0.05 compared with the WT+Con A group; ^#^*P* < 0.05 compared with the TPH1-/-+Con A group by one-way ANOVA and LSD test.

Oxidative stress is an important manifestation of hepatocyte injury induced by Con A. In Con A-treated mice, there is an imbalance between the oxidant and antioxidant status in liver tissue [8]. In our study, at 24 h after injection of Con A, levels of MDA, MPO, and NO were statistically significantly upregulated and activities of SOD and GSH were downregulated in liver tissue. Lack of serotonin protected hepatocyte against oxidative stress injury, characterized by dramatically inhibiting oxidative markers MDA (1.0 ± 0.3*vs*. 1.7 ± 0.5 nmol/mg.prot, *P* = 0.025), MPO (0.6 ± 0.2*vs*. 1.6 ± 0.5 U/g.prot, *P* = 0.006) and NO (0.8 ± 0.2*vs*. 1.7 ± 0.4 *μ*mol/g.prot, *P* = 0.001) and enhancing the activity of GSH (8.2 ± 0.7*vs*. 6.1 ± 1.3 mgGSH/g.prot, *P* = 0.016) in the liver after Con A injection (Figure 4). The recovery of serotonin by the supplement of 5-HTP exacerbated oxidative stress-mediated cellular injury in TPH1-/- ALI model mice.

### 3.5. Effect of Serotonin on Hepatocyte Apoptosis and Autophagy in Con A-Treated Mice

**Figure 5:**
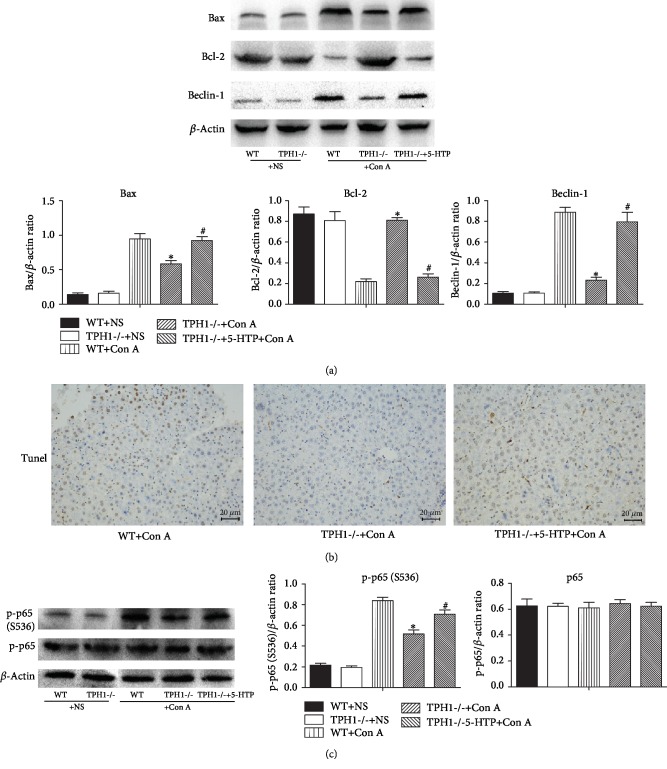
Serotonin induced apoptosis and autophagy of hepatocyte and activated NF-*κ*B in Con A-mediated ALI mice (24 h after the administration of Con A). (a) The hepatic protein expression levels of Bax, Bcl-2, and Beclin-1 were detected by Western blot, and the relative band intensities (fold of the sham group) were shown. (b) TUNEL assay was performed on liver tissue sections. (c) The protein levels of p65 and p-p65 were detected in liver tissue, and the relative band intensities (fold of the sham group) were shown. ^∗^*P* < 0.05 compared with the WT+Con A group; ^#^*P* < 0.05 compared with the TPH1-/-+Con A group by one-way ANOVA and LSD test.

In addition to necrosis, apoptosis and autophagy are also important pathways of cell death. We showed that, after the administration of Con A, the expressions of proapoptotic protein Bcl-2-asociated X protein (Bax) and proautophagic protein Beclin-1 significantly increased, and the expression of antiapoptotic protein B-cell lymphoma-2 (Bcl-2) significantly decreased in liver tissue. In addition, compared with the WT model group, the expressions of Bax and Beclin-1 decreased and Bcl-2 increased in the TPH1-/- model group (Figure 5(a)). However, pretreatment with 5-HTP significantly induced apoptosis and autophagy. TUNEL staining was performed in liver tissues of the three model groups. It was further suggested that serotonin could induce apoptosis of hepatocyte in Con A-mediated liver injury (Figure 5(b)).

### 3.6. Effect of Serotonin on the Activity of Nuclear Factor Kappa B (NF-*κ*B) in Con A-Treated Mice

NF-*κ*B is one of the families of transcriptional factor proteins, which participates in inflammatory responses, oxidative stress, and apoptosis and regulates a variety of inflammatory factors such as IL-2, IL-6, and TNF-*α* [28–30]. The protein expression of NF-*κ*B was further detected in ALI mice, and the result showed that intrahepatic p-p65 was markedly elevated after Con A injection. Significantly decreased expression of p-p65 was observed in the TPH1-/- model group compared with the WT model group (Figure 5(c)). However, administration of 5-HTP upregulated the expression of p-p65 in Con A-mediated liver injury, suggesting that serotonin may regulate inflammatory response and aggravate liver injury via activating intrahepatic NF-*κ*B.

### 3.7. Effect of Serotonin on Intrahepatic TLR Signaling Pathway in Con A-Treated Mice

**Figure 6:**
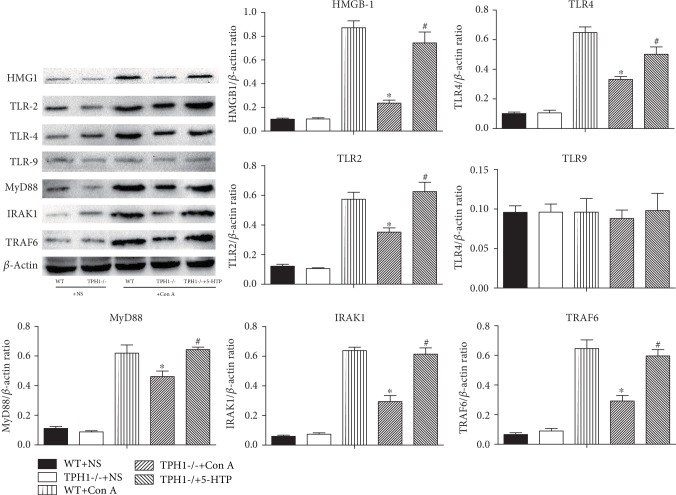
Serotonin induced the upregulation of HMGB1-TLRs and downstream molecules in Con A-mediated ALI mice. The protein expression levels of HMGB1, TLR2, and TLR4 and downstream molecules MyD88, IRAK1, and TRAF6 were detected in liver tissue by Western blot, and the relative band intensities (fold of the sham group) were shown. ^∗^*P* < 0.05 compared with the WT+Con A group; ^#^*P* < 0.05 compared with the TPH1-/-+Con A group by one-way ANOVA and LSD test.

As one of the earliest discovered pattern recognition receptors, TLRs are involved in inflammatory response and NF-*κ*B activation [28, 31]. Moreover, Con A stimulates the expression of TLR2, TLR4, and TLR9 and downstream pathways in liver tissue, resulting in the release of numerous proinflammatory factors [12, 13]. We then investigated whether serotonin affects TLR signaling pathway in Con A-induced hepatitis. As shown in Figure 6, Con A significantly upregulated the expressions of high mobility group protein B1 (HMGB1), TLR2, and TLR4 and downstream molecules myeloid differentiation factor 88 (MyD88), IL-1R-associated kinase 4 (IRAK1), and tumor necrosis factor receptor-associated factor 6 (TRAF6), while it had no effect on the expression of TLR9. However, deficiency of serotonin resulted in significant decreases in hepatic protein levels of HMGB1, TLR2, and TLR4 and downstream molecules in Con A-induced liver injury. The administration of 5-HTP significantly upregulated the expression of HMGB1, TLR2, and TLR4 and downstream molecules in Con A-treated TPH1-/- mice. These results suggested that serotonin may regulate NF-*κ*B activation and inflammatory response by activating the HMGB-TLR signaling pathway in Con A-induced hepatitis.

### 3.8. Effects of 5-HT_2A_ Receptor Antagonist Ketanserin in Con A-Treated Mice

**Figure 7:**
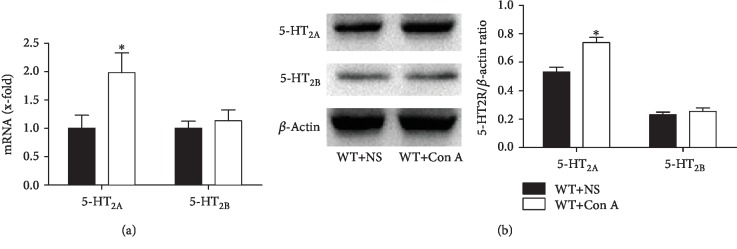
Serotonin may aggravate Con A-mediated ALI by binding to 5-HT_2A_ receptor. (a) The mRNA expressions of 5-HT_2A_ and 2B receptors in liver tissue were detected by qRT-PCR after the administration of Con A. (b) The protein expression levels of 5-HT_2A_ and 2B receptors were detected in liver tissue by Western blot, and the relative band intensities (fold of the sham group) were shown. ^∗^*P* < 0.05 compared with the WT+NS group by Wilcoxon test.

Among serotonin receptors, 5-HT_2A_ and 2B receptors are relatively highly expressed in liver tissue and are closely related to the pathophysiology of several liver diseases [32]. After injection of Con A, significant increases in intrahepatic protein and mRNA levels of 5-HT_2A_ receptor, but not 5-HT_2B_ receptor, were observed (Figure 7). It was suggested that serotonin may aggravate liver injury by binding to intrahepatic 5-HT_2A_ receptor.

**Figure 8:**
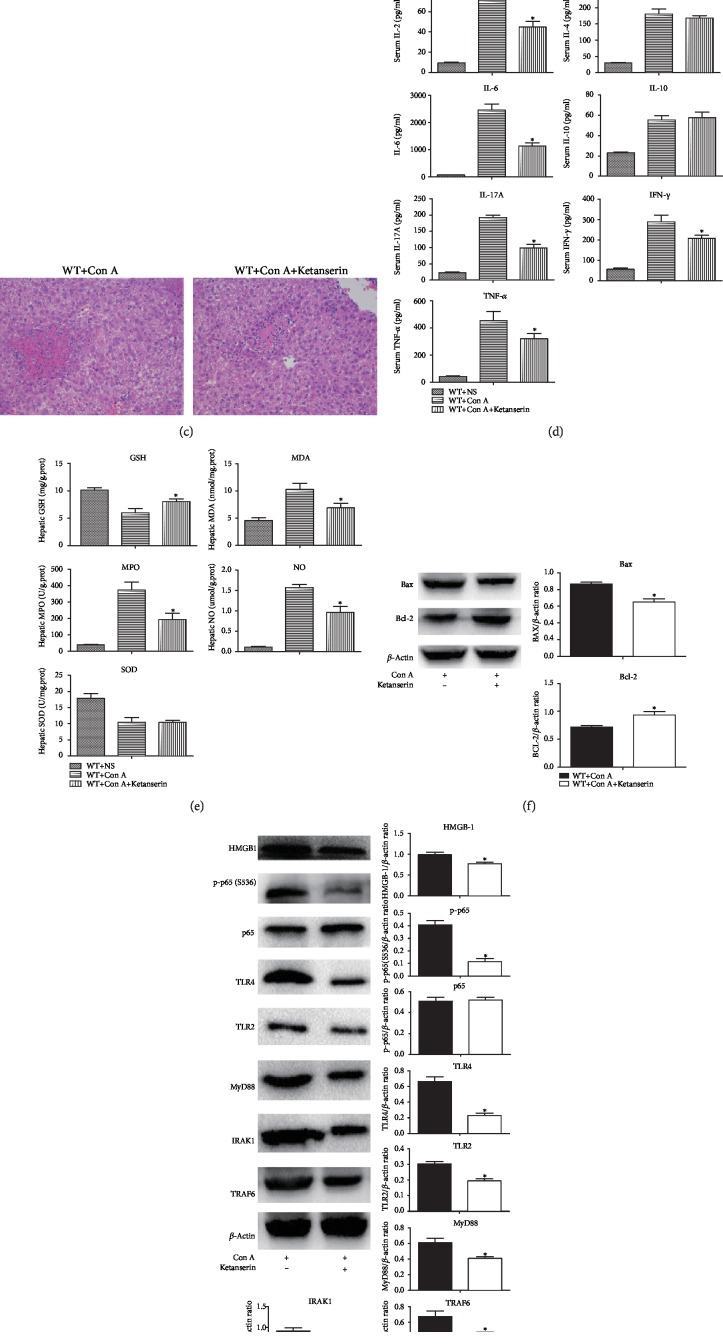
5-HT_2A_ receptor antagonist Ketanserin attenuates Con A-mediated ALI. (a) The protein expression levels of 5-HT_2A_ were detected in liver tissue by Western blot, and the relative band intensities (fold of the sham group) were shown. (b) Comparison of serum ALT and AST in ALI mice. (c) H&E staining (×200) was performed on liver tissue slices at 24 h after the administration of Con A. (d) The levels of serum inflammatory cytokines were assessed by ELISA at 24 h after the administration of Con A. (e) The levels of oxidative stress indicators in liver tissue were assessed at 24 h after the administration of Con A. (f) The hepatic protein expression levels of Bax and Bcl-2 were detected by Western blot, and the relative band intensities (fold of the sham group) were shown. (g) The protein expression levels of HMGB1-TLRs and downstream molecules were detected in liver tissue by Western blot, and the relative band intensities (fold of the sham group) were shown. ^∗^*P* < 0.05 compared with the control group by Wilcoxon test.

Ketanserin can significantly inhibit the expression of 5-HT_2A_ receptor in peripheral tissues [33]. As shown in Figure 8(a), after intraperitoneal injection of Ketanserin in mice, a significant decrease in the intrahepatic expression of 5-HT_2A_ receptor was observed. In Con A-induced liver injury, Ketanserin significantly reduced serum levels of transaminase and improved liver pathological damage (Figures 8(b) and 8(c)). This protective effect may be related to the improvements of inflammatory response (IL-2, IL-6, IL-17A, IFN-*γ*, and TNF-*α*), oxidative stress damage (GSH, MDA, MPO, and NO), and hepatocyte apoptosis (Bax and Bcl-2) (Figures 8(d)–8(f)). In addition, the HMGB1-TLR signaling pathway was involved in the protective role of Ketanserin in Con A-induced hepatitis (Figure 8(g)).

## 4. Discussion

Serotonin is involved in the progresses of a variety of liver diseases. Recent studies have indicated that serotonin plays a double-edged role in the pathophysiological processes of the liver. On the one hand, serotonin could stimulate liver regeneration after hepatectomy [34]. On the other hand, serotonin may aggravate the progress of nonalcoholic fatty liver, liver fibrosis, and hepatocellular carcinoma [22–24].

**Figure 9:**
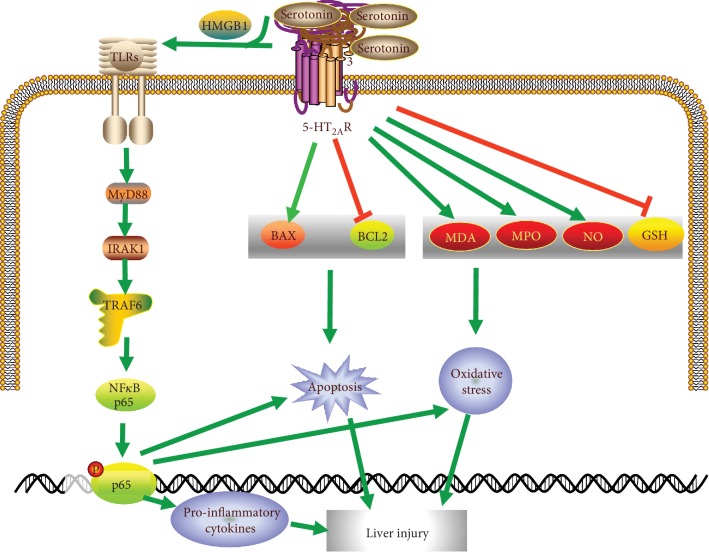
Illustration for the mechanism underlying serotonin for the aggravation of Con A-induced ALI.

This study is the first to investigate the potential function of serotonin in Con A-induced ALI. We found that the lack of serotonin significantly attenuated Con A-induced hepatitis and supplement of serotonin resulted in higher sensitivity to liver damage. These data suggest that serotonin is critically involved in the pathogenesis of Con A-mediated liver injury. Mechanistic studies further revealed that serotonin functions as a positive regulator in inflammation response, oxidative stress injury, and hepatocyte apoptosis via stimulating the release of HMGB1 and activating the TLR signaling pathway in the Con A-induced ALI model (Figure 9). Furthermore, inhabitation of 5-HT_2A_ receptor protected against Con A-induced liver injury.

The injection of Con A can trigger a severe inflammatory response characterized by the release of numerous inflammatory factors via recruiting leukocytes [35]. We found that the lack of serotonin significantly inhibited the release of proinflammatory factors both in serum and in liver tissue, suggesting that serotonin may aggravate Con A-induced ALI by promoting inflammation response.

Oxidative stress has been considered as a cornerstone in the pathophysiological process of various hepatic diseases. In Con A-induced liver injury, one of the important characteristics is the imbalance of oxidative stress [36]. He et al. showed elevated oxidative products (MDA and 8-hydroxy-2′-deoxyguanosine) and reduced activities of SOD and catalase in Con A-treated mice [7]. El-Agamy reported that the administration of Con A markedly increased hepatic MDA content and decreased hepatic levels of GSH and SOD compared to the control group [8]. The present study indicated that Con A injection caused a marked elevation of lipid peroxidation as presented by excessive accumulation of MDA, MPO, and NO in liver tissue. In addition, there was a significant inhibition in the antioxidant status indicated by decreased GSH and SOD activities.

In recent years, several studies have explored the effects of serotonin in oxidative stress. In 2007, Nocito et al. established the mice model of fatty liver and found that serotonin deficiency reduced intrahepatic MDA [37]. Via the establishment of colitis model, Mousavizadeh et al. found that tropisetron (serotonin receptor antagonist) reduced the activities of MPO and MDA in colon tissue [38]. Fluoxetine, a serotonin receptor inhibitor, could improve hepatic oxidative stress injury [39]. In addition, serotonin regulates postoperative intra-abdominal adhesion formation by facilitating oxidative stress [40]. Serotonin also increases the intrahepatic level of MDA and aggravates multiple organ dysfunction syndrome induced by zymosan [26]. In our study, however, serotonin deficiency or Ketanserin pretreatment induced significant suppression of lipid peroxidation and restoration of antioxidant status in Con A-treated mice.

Apoptosis and autophagy have been reported to play controversial roles in Con A-treated mice. Feng et al. showed that the relative protein and mRNA expressions of Bax, caspase-3, caspase-9, and Beclin-1 were significantly enhanced after Con A treatment [41]. TUNEL assay further demonstrated that positive areas of apoptotic cells were significantly larger in Con A-treated mice [41]. Serotonin could activate cell apoptosis and induce the expression of autophagy-related effectors LC3, Beclin-1, and ATG3 [42, 43]. 5-HT_2B_ receptor blocker SB-204741 inhibited apoptotic signaling pathway [44]. We demonstrated that serotonin aggravated Con A-induced ALI by upregulating the expressions of Bax and Beclin-1 and downregulating Bcl-2.

Recent studies have shown that the administration of Con A stimulates the intrahepatic expressions of TLRs [45, 46]. As one of the earliest and most widely studied TLRs, TLR4 triggers a cascade of downstream signals such as NF-*κ*B and induces the release of numerous proinflammatory cytokines. TLR4 can activate MyD88 and form the complex of TLR4/MyD88. The complex further activates IRAK4 and enables NF-*κ*B to enter into the nucleus. Pomytkin et al. showed that intestinal serotonin could activate TLR4 [47]. Fluoxetine, a serotonin reuptake inhibitor, inhibits the TLR signaling pathway and presents an anti-inflammatory property [48]. In our study, Con A significantly upregulated the expressions of TLR2 and TLR4 and downstream molecules and activated NF-*κ*B, while deficiency of serotonin attenuated the influence. HMGB1, which is involved in Con A-treated mice, is acknowledged as an important ligand of TLRs [49–51]. We demonstrated that serotonin could stimulate the release of HMGB1, suggesting that serotonin may regulate inflammation response and aggravate liver injury via activating HMGB1-TLR signaling pathways in Con A-treated mice.

The role of serotonin is largely determined by serotonin receptors. There are mainly 5-HT_2A_ and 2B receptors on the surface of hepatocytes. Via acting on the two receptors, peripheral serotonin produces reactive oxygen species and lipid peroxide and then increases hepatocyte injury through mitochondrial damage and inflammatory stimulation. It has been reported that serotonin can be involved in the pathophysiological process of liver regeneration via 5-HT_2B_ receptor [52], hepatic steatosis via 5-HT_2A_ and 2B receptors [32], viral hepatitis via 5-HT_2A_ receptor [53], liver fibrosis via 5-HT_2A_ receptor [23], and liver cancer via 5-HT_2B_ receptor [54]. We found that the intrahepatic expression of 5-HT_2A_ receptor was increased in Con A-treated mice. We further suggested that, via binding to 5-HT_2A_ receptor, serotonin activated inflammatory response, promoted oxidative stress injury, and ultimately aggravated Con A-mediated ALI.

Therefore, we speculate that serotonin promotes inflammation response, oxidative stress injury, and hepatocyte apoptosis via activating the HMGB1-TLR signaling pathway and binding to 5-HT_2A_ receptor and eventually aggravates Con A-induced ALI. The depletion of serotonin or the application of 5-HT_2A_ receptor inhibitor may protect mice from liver damage. Serotonin provides the clues for basic and clinical investigations of ALI and may become a new potential target for therapy.

## Data Availability

The data used to support the findings of this study are available from the corresponding authors upon request.

## Supplementary Materials

Supplementary Materials Supplementary Figure 1: H&E staining (×200) was performed on liver tissue sections at 24 h after the administration of NS or different dosages of Con A (8, 15, and 25 mg/kg body weight). Supplementary Figure 2: serum levels of basal serotonin (without the administration of Con A or NS) in WT mice and in TPH1-/- mice with or without the supplement of 5-HTP. ^∗^*P* < 0.05 compared with WT mice; ^#^*P* < 0.05 compared with TPH1-/- mice. Supplementary Table 1: upstream and downstream primers. Supplementary Table 2: antibodies used for WB analysis.
