# Supplementary material for: The Role of Serotonin in Concanavalin A-Induced Liver Injury in Mice
Source: Oxid Med Cell Longev. 2020 Jan 4;2020:7504521. doi: 10.1155/2020/7504521 (PMC6969644; doi:10.1155/2020/7504521)
Supplement: Supplementary Materials — Supplementary Figure 1: H&E staining (×200) was performed on liver tissue sections at 24 h after the administration of NS or different dosages of Con A (8, 15, and 25 mg/kg body weight). Supplementary Figure 2: serum levels of basal serotonin (without the administration of Con A or NS) in WT mice and in TPH1-/- mice with or without the supplement of 5-HTP. ∗P < 0.05 compared with WT mice; #P < 0.05 compared with TPH1-/- mice. Supplementary Table 1: upstream and downstream primers. Supplementary Table 2: antibodies used for WB analysis. [file 7504521.f1.docx]

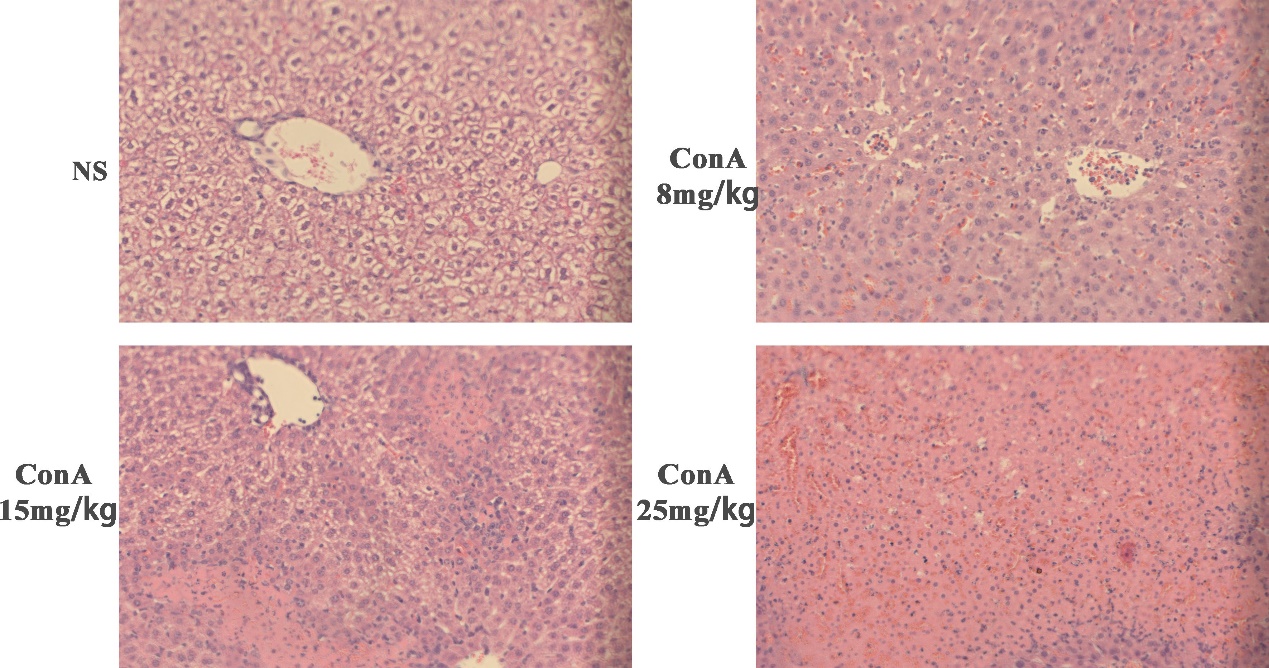


Supplementary FIGURE 1: H&E staining (× 200) was performed on liver tissue sections at 24h after the administration of NS or different dosages of con A (8, 15, and 25mg/kg body weight).


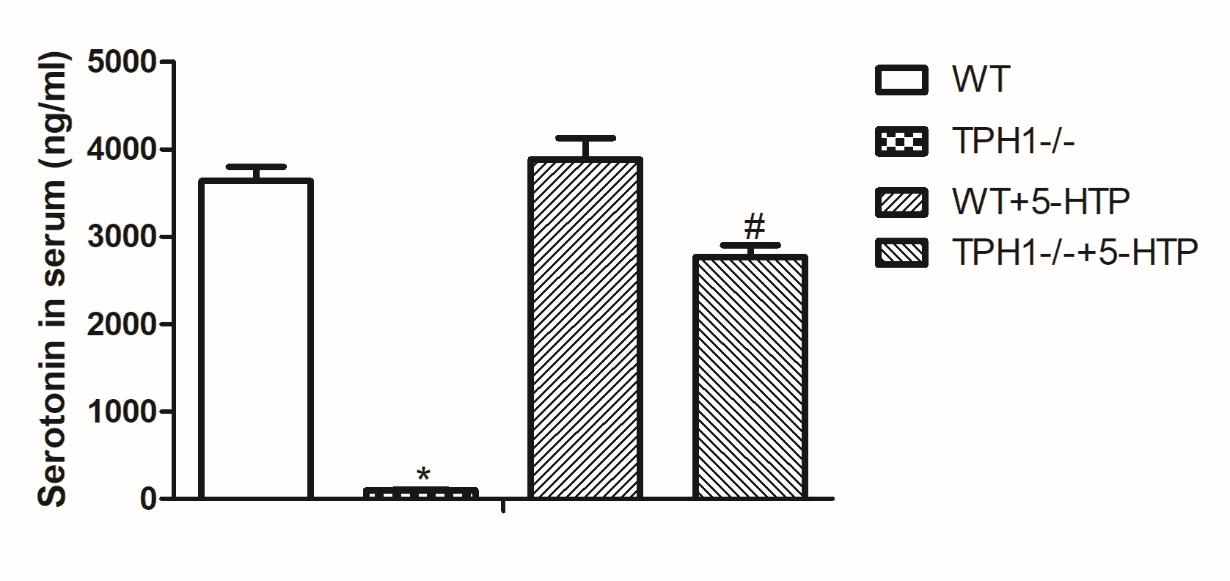


Supplementary FIGURE 2: Serum levels of basal serotonin (without the administration of Con A or NS) in WT mice and in TPH1-/- mice with or without the supplement of 5-HTP. ^*^*P* < 0.05 compared with WT mice; ^#^*P* < 0.05 compared with TPH1-/- mice.

Supplementary TABLE 1: Upstream and downstream primers.

| Gene | Primers(5′-3′) |
| --- | --- |
| IL-2 | Forward: TCTGCGGCATGTTCTGGATT  Reverse: TGTGTTGTCAGAGCCCTTTAGT |
| IL-4 | Forward: CGCCATGCACGGAGATG  Reverse: CGAGCTCACTCTCTGTGGTGTT |
| IL-6 | Forward: TCCATCCAGTTGCCTTCTTG  Reverse: TTCCACGATTTCCCAGAGAAC |
| IL-10 | Forward: TTTGAATTCCCTGGGTGAGAA  Reverse: GGAGAAATCGATGACAGCGC |
| IL-17A | Forward: ATTATGGGTGGTGAGAGCCG  Reverse: GTTCCTGTCATTTTGTCCAATTCA |
| IFN-γ | Forward: CCTCAAACTTGGCAATACTCA  Reverse: CTCAAGTGGCATAGATGTGGA |
| TNF-α | Forward: AGGTACAACCCATCGGCTGG  Reverse: GGTCTGGGCCATAGAACTGA |
| 5-HT_2A_ | Forward: CGAAGCCTCGAACTGGACAAT  Reverse: CCGCAATGGTGAGAATAATCACG |
| 5-HT_2B_ | Forward: ACCTGATCCTGACTAACCGTT  Reverse: TGGGTATTATCACCGCGAGTAT |
| GAPDH | Forward: GCCGCCTGGAGAAACCTGCCAAGT  Reverse: TATTCAAGAGAGTAGGGAGGGCTC |

Supplementary TABLE 2: Antibodies used for WB analysis

| Antibody | Purchased from |
| --- | --- |
| Bcl-2 | Cell Signaling Technology |
| Bax | Cell Signaling Technology |
| Beclin-1 | Cell Signaling Technology |
| β-actin | Cell Signaling Technology |
| NF-κB p65 | Cell Signaling Technology |
| NF-κB p-p65 S536 | Cell Signaling Technology |
| TLR2 | Cell Signaling Technology |
| TLR4 | Cell Signaling Technology |
| TLR9 | Cell Signaling Technology |
| HMGB1 | Cell Signaling Technology |
| MyD88 | Cell Signaling Technology |
| IRAK1 | Cell Signaling Technology |
| TRAF6 | Cell Signaling Technology |
| 5-HT_2A_ receptor | Cell Signaling Technology |
| 5-HT_2B_ receptor | Cell Signaling Technology |
